# Roles of Reconstituted High-Density Lipoprotein Nanoparticles in Cardiovascular Disease: A New Paradigm for Drug Discovery

**DOI:** 10.3390/ijms21030739

**Published:** 2020-01-23

**Authors:** Jiansheng Huang, Dongdong Wang, Li-Hao Huang, Hui Huang

**Affiliations:** 1 Department of Medicine, Vanderbilt University Medical Center, 318 Preston Research Building, 2200 Pierce Avenue, Nashville, TN 37232, USA; 2 Institute of Clinical Chemistry, University Hospital Zurich, Wagistrasse 14, 8952 Schlieren, Switzerland; dongdong.wang@univie.ac.at; 3 Pathology and Immunology Department, Washington University School of Medicine, St. Louis, MO 63110-1093, USA; paul.huang@wustl.edu; 4 Department of Biochemistry, Vanderbilt University, Nashville, TN 37232, USA; hui.huang@vanderbilt.edu

**Keywords:** apolipoproteins and inflammatory properties, ABCA1, reconstituted high-density lipoprotein, cardiovascular disease, molecular imaging

## Abstract

Epidemiological results revealed that there is an inverse correlation between high-density lipoprotein (HDL) cholesterol levels and risks of atherosclerotic cardiovascular disease (ASCVD). Mounting evidence supports that HDLs are atheroprotective, therefore, many therapeutic approaches have been developed to increase HDL cholesterol (HDL-C) levels. Nevertheless, HDL-raising therapies, such as cholesteryl ester transfer protein (CETP) inhibitors, failed to ameliorate cardiovascular outcomes in clinical trials, thereby casting doubt on the treatment of cardiovascular disease (CVD) by increasing HDL-C levels. Therefore, HDL-targeted interventional studies were shifted to increasing the number of HDL particles capable of promoting ATP-binding cassette transporter A1 (ABCA1)-mediated cholesterol efflux. One such approach was the development of reconstituted HDL (rHDL) particles that promote ABCA1-mediated cholesterol efflux from lipid-enriched macrophages. Here, we explore the manipulation of rHDL nanoparticles as a strategy for the treatment of CVD. In addition, we discuss technological capabilities and the challenge of relating preclinical in vivo mice research to clinical studies. Finally, by drawing lessons from developing rHDL nanoparticles, we also incorporate the viabilities and advantages of the development of a molecular imaging probe with HDL nanoparticles when applied to ASCVD, as well as gaps in technology and knowledge required for putting the HDL-targeted therapeutics into full gear.

## 1. Introduction

Epidemiological studies identified several independent risk factors for cardiovascular disease (CVD), including hypertension, age, smoking, insulin resistance, elevated low-density lipoprotein cholesterol (LDL-C) levels, and triglyceride levels [1]. The majority of people establish plaques during young adulthood, making plaque regression the optimal therapeutic strategy [2,3,4,5]. The most effective LDL lowering agent, PCSK9 inhibitor evolocumab, only regressed coronary atheroma volume as assessed by serial coronary intravascular ultrasound by 0.95%, although 78 weeks of treatment reduced the LDL-C to 36.6 mg/dL in humans [6,7]. Clinical studies confirm that apolipoprotein AI (apoAI) can largely promote the regression of atherosclerosis by increasing functional high-density lipoprotein (HDL) particles [8,9,10,11,12].

## 2. Atheroprotective Functions of HDL

Additional evidence for the atheroprotective effects of apoAI or HDL derives from mouse models of experimental atherosclerosis and clinical trials. One of the most effective approaches is to promote new HDL particle formation by enhancing endogenous production of apoAI, which is supported by using human apoAI transgenic mice studies as well as virus-mediated overexpression of apoAI in a mouse model of experimental atherosclerosis [13,14,15]. In addition, RVX-208, a bromodomain and extraterminal domain (BET) protein inhibitor, is able to increase the production of apoAI [14,16,17]. A recent study found that RVX-208 significantly reduces major adverse cardiovascular events (MACE) in diabetes patients with CVD although administration of RVX-208 did not promote the regression of coronary atheroma due to its moderate effect on raising HDL-C levels in patients with CVD [17,18]. Similarly, RVX-208 treatment decreased circulating levels of vascular inflammation mediators, such as adhesion molecules, tumor necrosis factor α (TNFα), and interleukin 1β I(L-1β) in CVD patients [19]. Inhibition of the inflammation-induced adhesion molecule gene expression by RVX-208 may contribute to MACE reduction in the phase III clinical trial. Therefore, the identification of novel molecules that regulate apoAI synthesis is essential for increasing apoAI and HDL production and improving vascular inflammation.

HDL protective functions, including anti-inflammatory and mediation of reverse cholesterol transport (RCT), likely contribute to the regression of established plaques and are central in protecting against CVD [20,21]. For example, HDLs can prevent LDL-induced monocyte migration and inhibit the expression of pro-inflammatory cytokines [22]. This anti-inflammatory property is partly due to HDL-associated proteins, such as platelet-activating factor acetylhydrolase and paraoxonase1 (PON1) [23,24]. Importantly, recent studies demonstrate that HDL-cholesterol (HDL-C) levels are inversely associated with risks of CVD [9,25,26,27]. In addition, it is reported that HDL function is impaired in familial hypercholesterolemic (FH) patients [28,29]. HDL recirculation through tissues is limited in experimental psoriasis, potentially leading to the development of atherosclerosis [30], and there is abundant dysfunctional apoAI in human atherosclerotic lesions [31,32]. Therefore, HDL-targeted therapy is a promising approach for the treatment of CVD.

Much progress has been made to increase HDL-C levels in plasma using orally-administered small molecules such as niacin and cholesteryl ester transfer protein (CETP) inhibitors [33,34]. Large-scale human clinical studies demonstrated that HDL-targeted therapy, such as niacin, failed to show a clinical benefit by raising HDL-C levels when combined with statin therapy (nicely reviewed in [20,33]). The development of CETP inhibitors has had a long road with three compounds either halted earlier or ineffective in phase III clinical trials. The latest REVEAL (randomized evaluation of the effects of anacetrapib through lipid modification) study showed that anacetrapib increased HDL-C levels and reduced coronary heart disease when combined with statin therapy [35]. The efficacy of anacetrapib seems to be partially due to the reduction in non-HDL-C, rather than an increase of HDL-C levels. On the other hand, these compounds may interfere with the lipid metabolism and cause alterations in HDL structure and HDL-C composition [36]. The changed composition of HDL subpopulations is directly related to their cardioprotective functions. HDLs have any combination of nearly 90 different proteins and the HDL-C measurement only provides the values of cholesterol or its ester within the HDL complex, and is only made up of 20% of the HDL [37,38]. There is no doubt that the atheroprotective functions of HDLs could change after raising cholesterol levels on the HDL complex. These HDL-C-raising trials that interrupted HDL catabolism to increase HDL-C, ultimately resulted in production of large cholesteryl ester-enriched particles. Compared to small dense human HDL3 particles, large HDL particles may not interact efficiently with the ATP binding cassette transporter A1 (ABCA1) and ABCG1 [36]. This turns out to be essential, given that the interaction with ABCA1 and ABCG1 plays an important role in promoting cholesterol efflux to HDLs from macrophages. In addition, pharmacologically raising HDL-C levels with niacin and CETP inhibitors may affect HDL subsets, cause an increase in cholesterol levels in HDLs, and increase the size of the HDL particle, which may counteract or offset the HDL’s beneficial effects [39,40,41]. Supporting these notions, a recent study demonstrated that CETP inhibitor gives rise to fatty liver and insulin resistance in CETP-expressing transgenic mice fed with a high-fat diet [42]. Recent studies provide solid evidence that HDL efflux capacity is inversely related to cardiovascular events after being adjusted with other risk factors, including HDL-C levels [26,27]. Therefore, studies to restore HDL functions are needed to increase the number of particles capable of promoting ABCA1-mediated cholesterol efflux.

## 3. Therapeutic Approaches Targeting Increasing HDL Particles

**Figure 1:**
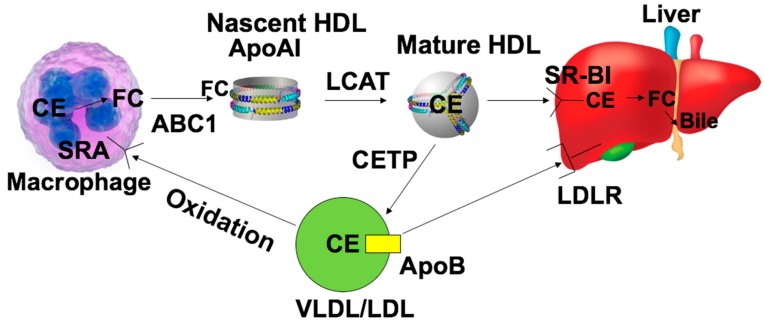
In vivo reverse cholesterol transport (RCT) pathway. Modified low-density lipoproteins (LDLs) are recognized by scavenger receptor A (SRA) and taken up by macrophages, which lead to the formation of a foam cell. Apolipoprotein AI (ApoAI) or nascent high-density lipoproteins (HDLs) promote the cholesterol efflux from foam cells by interacting with ATP-binding cassette transporters (ABC1) ABCA1 or ABCG1. Free cholesterol in HDLs is esterified into cholesteryl ester (CE) by lecithin cholesterol acyltransferase (LCAT) to produce the mature HDL. Liver scavenger receptor class B type I (SR-BI) is responsible for recognition of HDLs and promotes the uptake of cholesterol. CE is de-esterified and converted into bile acid, which is secreted into the bile. In humans, CE can be transported onto ApoB by cholesterol ester transport protein (CETP) and leads to the formation of LDLs or very low-density lipoproteins (VLDLs).

**Figure 2:**
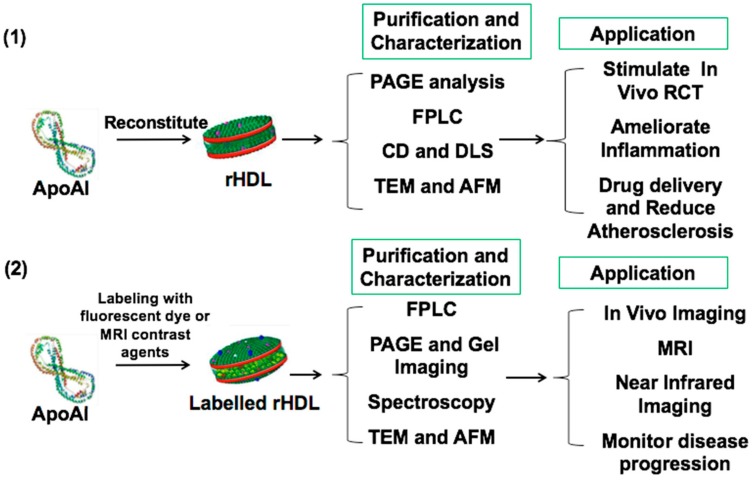
Synthesis and characterizations of rHDL and rHDL imaging probes. rHDLs are made up of reconstituted apoAI and phospholipids. rHDLs are characterized by biochemical approaches such as polyacrylamide gel electrophoresis (PAGE) analysis, and transmission electron microscopy (TEM) or atomic force microscopy (AFM) for measuring dimensions of rHDL nanoparticles after purification with fast protein liquid chromatography (FPLC). The biophysical properties of the rHDL particles is determined by using dynamic light scattering (DLS) and circular dichroism (CD) spectroscopy. rHDLs can be labeled with fluorescent dye or MRI imaging contrast agents for in vivo imaging purposes.

Recent failures of CETP inhibition aimed at increasing HDL-C levels to decrease the risk of CVD have shifted the focus of HDL research towards a better understanding of mechanisms to preserve HDL function(s), rather than raising HDL-C levels. HDLs mediate the transport of cholesterol from peripheral tissues to the liver from which it can be secreted as bile acid, the process of which is known as reverse cholesterol transport (RCT). There are basically three steps in this process: (1) cholesterol efflux, where apoAI and HDLs interact with ABCA1 and ABCG1 to remove excess cholesterol from lipid-enriched macrophages as shown in Figure 1; (2) lipoprotein remodeling after accepting cholesterol and HDLs with structural modifications; and (3) cholesterol uptake by scavenger receptor class B type I (SR-BI) expressed in the liver, where cholesterol is converted into bile acid for the final excretion into bile and feces [43,44].

## 4. ApoAI Mimetic Peptide

Recent studies suggest that apoAI mimetic peptides serve as a promising new therapeutic approach by promoting the formation of pre-β HDLs, increasing cholesterol efflux, and preventing lipoprotein oxidation [45]. The sequence of 18A (D-W-l-K-A-F-Y-D-K-V-A-E-K-l-K-E-A-F) does not have any sequence homology to apoAI, but has the ability to display a class A amphipathic helix similar to those found in apoAI, which is the determinant of the lipid-binding properties. Among the most extensively studied apoAI mimetic peptides is the 4F peptides (Ac-D-W-F-K-A-F-Y-D-K-V-A-E-K-F-K-E-A-F-NH2). ApoAI mimetic 4F peptides were synthesized from both all D-amino acids (D-4F) and all L-amino acids (L-4F). The apoAI mimetic 4F peptide exhibited a remarkable binding affinity for oxidized lipids compared to the wild type apoAI [46]. Treatment with D-4F increased RCT from macrophages in Apoe^−/−^ mice [47]. In addition, Rev-D4F was developed to determine its effect on atherosclerosis. Interestingly, Rev-D4F reduced the atherosclerotic lesion and macrophage content without altering the HDL-cholesterol levels in Apoe^−/−^ mice. Rev-D4F also prevented LDL oxidation, reduced the expressions of endothelial cell vascular cell adhesion molecule 1 (VCAM-1) and monocyte chemotactic factor 1 (MCP-1), and improved vascular inflammation in Apoe^−/−^ mice [48]. More importantly, the recent randomized controlled clinical trial demonstrated that oral apoAI mimetic D-4F reduced the HDL inflammatory index in high-risk patients [49]. Taken together, these results support the development of apoAI mimetic peptides as a therapeutic approach for the treatment of atherosclerosis.

## 5. Reconstituted High-Density Lipoprotein (rHDL) Nanoparticles

Proteomic studies found HDLs contain hundreds of proteins and lipids [10]. Therefore, HDLs are highly heterogeneous. Using rHDLs as a tool allows us to better understand HDL composition and its roles in CVD. rHDLs are synthesized by the combination of apoAI and phospholipids (Figure 2). The biochemical properties of the rHDL particles are determined using transmission electron microscopy (TEM), atomic force microscopy (AFM), dynamic light scattering (DLS), and circular dichroism (CD) spectroscopy after purification with fast protein liquid chromatography (FPLC) [50]. It is reported that three different apoAI have been utilized to synthesize rHDLs, including human apoAI de-lipidated from HDLs, recombined human apoAI purified from *Escherichia coli*, and synthesized apoAI peptides. Development of rHDL nanoparticles that mimic pre-β HDLs, could promote cholesterol efflux from lipid-enriched macrophages, thereby reducing atherosclerosis. Many different rHDL nanoparticles (apoAI-bound phospholipid disks or delipidated HDL particles, mutant apoAI proteins, and apoAI mimetic peptides) have been constructed and investigated in animal studies and human clinical trials [51].

## 6. Reconstituted ApoAI Milano/Palmitoyl-Oleoyl Phosphatidyl Choline (POPC)

Emerging therapeutic approaches targeting rHDL have been investigated to reduce the risk of cardiovascular events in the past decades [52,53]. One of the important therapies is apoAI Milano (apoA-IM), a naturally occurring mutant of apoAI that was shown to be related with cardioprotective effects [54]. Therefore, the first human recombinant apoA-IM ETC-216 was developed by the combination of the recombinant apoAI Milano with POPC (mass ratio is 1:1.1). Preclinical studies demonstrated that the recombinant apoA-IM increased cholesterol efflux from macrophages and RCT, and reduced atherosclerotic plaques in mice [55]. Interestingly, the recombinant HDLs (Milano) exert greater anti-inflammatory and plaque stabilizing properties than wild-type HDLs [56]. In vitro studies demonstrated that the apoA-IM protein dramatically inhibited expression of cycloxygenase (COX)-2 and MCP-1 in macrophages compared to wild-type apoAI [57]. Further, infusion of recombinant apoAI Milano-phospholipid complexes causes rapid regression of atherosclerosis. Infusion of rHDLs (Milano) significantly induce a greater aortic plaque regression compared to wild-type HDL treatment in a rabbit model examined by magnetic resonance imaging (MRI) [57]. More importantly, infusion of ETC-216 was related to a significant reduction of the atherosclerotic plaque in patients with acute coronary syndrome (ACS) [56]. Unfortunately, the clinical trial was discontinued because a serious adverse reaction (dose-dependent increases in neutrophils and decreases in lymphocytes) occurred due to small quantities of residual host cell proteins (HCPs).

## 7. MDCO-216

To overcome the adverse effects of ETC-216, another recombinant apoA-IM, MDCO-216, was developed and its efficacy was examined in patients with coronary artery disease [58,59]. MDCO-216 infusion reduced small HDLs and resulted in the production of α-1 and α-2 HDLs containing both wild-type apoAI and apoAI Milano. Administration of MDCO-216 (ApoA-1 Milano/POPC) promoted ABCA1-mediated cholesterol efflux and pre-β HDLs in healthy volunteers and patients with stable coronary artery disease [60,61]. Subsequently, HDL mimetic containing the recombinant apoAI Milano (five weekly infusion of MDCO-216) was developed to determine its effect on coronary disease in patients with an acute coronary syndrome in the MILANO-PILOT trial [62]. However, administration of MDCO-216 failed to promote the regression of the atherosclerotic plaque when combined with statin therapy [62].

## 8. CER-001

CER-001 was made by recombinant human apoAI, sphingomyelin (SPM), and dipalmitoylphosphatidyl glycerol (DPPG) (protein to phospholipid = 1:2.7). CER-001 increased the reverse lipid transport and promoted atherosclerosis regression in Ldlr^−/−^ mice. More importantly, there was a significant reduction in macrophage content and a reduction in VCAM-1 expression in the plaque after treatment of mice with CER-001 [63]. However, six weekly infusions of HDL-mimetic agent CER-001 did not regress atherosclerosis measured by intravascular ultrasonography (IVUS) [64]. Similarly, in another trial, 10 weekly infusions of CER-001 failed to promote the regression of coronary atherosclerosis in statin-treated patients with ACS and high plaque burden [65].

## 9. CSL-111

CSL-111, a reconstituted HDL particle, is combined by native apoAI and phospholipids. Recent studies demonstrate that rHDL CSL-111 infusion reduced the total number of circulating leucocytes and neutrophils in mice following ischemic reperfusion (I/R) [66,67]. CSL-111 reduced the number of circulating monocytes and B lymphocytes and improved post-ischemic heart function through the modulation of acute inflammatory response [66,68]. rHDL reduced the percentage of B lymphocytes recruited into the heart of mice on a high-fat diet (HFD), stressing its remarkable anti-inflammatory function. However, in a human clinical study, CSL-111 failed to reduce the volume of atherosclerotic plaque, but it did improve the plaque characterization index and coronary score by quantitative coronary angiography (QCA), thereby suggesting therapeutic potential [69]. CSL-111 was halted from development due to the increase of liver enzymes [69].

## 10. CSL-112

A new formulation of human rHDL CSL-112 was developed by reconstituting human plasma-derived apoAI with phosphatidylcholine to form disc-shaped HDL particles. There are two molecules of human apoAI, about 110 molecules of phosphatidylcholine and sucrose as a stabilizing agent. CSL-112 significantly increased the cholesterol efflux from J774 macrophages [70]. Infusion of CSL-112 was shown in several studies to modify plaque characterization on IVUS [69,71]. CSL-112 increased RCT and modified plaque lipid composition. These results support the continued evaluation of the safety and efficacy of CSL-112 as a new therapy for patients with ACS. However, larger populations are needed to determine the clinical efficacy of CSL-112 in ACS patients with a higher risk of adverse events. A large phase III study (NCT03473223) is currently recruiting patients and is expected to be concluded in 2022. Whether or not a long-term study of CSL-112 in large populations is able to reduce CAD risk will be determined.

These major rHDL-based therapies (MDCO-216, CER-001, and CSL-112) are remarkably different in composition, dosing, pharmacokinetics, and pharmacodynamics as shown in Table 1. There may be many multifactorial reasons for the failure of the rHDL-targeted therapy. First of all, the duration of the study could have been too short. In addition, the dosage could have been too low to be effective or not frequent enough; thus, potentially beneficial effects of administration of rHDLs in the long term could have been neglected. Moreover, it is known that oxidative stress and inflammation readily change HDL composition and function [72]. Post-translational modifications, phospholipid depletion, and enrichment with proinflammatory proteins like serum amyloid A (SAA) or myeloperoxidase (MPO) may change its atheroprotective function after administration of rHDLs [73,74,75]. Moreover, the phosphatidylcholine moiety of the rHDL nanoparticles may be immediately degraded in plasma, generating lysophospholipids that might alter the functions of HDLs [36].

## 11. rHDL Nanoparticles as a Drug Delivery Vehicle

The application of rHDL nanoparticles for delivering therapeutic compounds for the treatment of cancer has been studied extensively [76,77,78]. Recent studies show that rHDL nanoparticle serve as a drug delivery system to deliver compounds efficiently into macrophages and atherosclerotic plaques [79]. To investigate the immunomodulatory drugs for atherosclerosis, several nanoparticles were developed to increase the specificity of the drug delivery. rHDLs were efficiently used to deliver a liver X receptors (LXR) agonist GW3965 to atherosclerotic plaques of Apoe^−/−^ mice [80]. Importantly, rHDLs loaded with GW3965 completely abolished the liver toxicity of GW3965 in a one-week intensive treatment regimen in atherosclerotic mice. The long-term treatment with rHDLs significantly reduced atherosclerotic plaques in Apoe^−/−^ mice [81].

Statins have potent anti-inflammatory functions, but these cannot be fully exploited with oral statin therapy owing to a low systemic bioavailability. Interestingly, an injectable rHDL nanoparticle was synthesized to deliver simvastatin, and the effect of simvastatin-rHDL on atherosclerotic plaques was examined in mice. This study demonstrates that statin-loaded reconstituted HDL nanoparticles improved inflammation in atherosclerotic plaque [82]. More interestingly, nanoparticle-based delivery of simvastatin inhibited plaque macrophage proliferation in Apoe^−/−^ mice with advanced atherosclerotic plaques [83]. rHDL nanoparticles increased the plasma half-life of statins to 20 h. In addition, a recent study showed that rHDL-mediated targeted delivery of the LXR agonist promoted atherosclerosis regression [84].

Arachidonic acid (AA) was engineered into the rHDL complex to increase the efficacy of statins. AA-LT-rHDL (arachidonic acid-lovastatin-rHDL) exhibited lower reactivity with LCAT and more potent inhibition effects on foam cell formation in the presence of LCAT because of less undesired LT leakage during the remodeling of rHDLs induced by LCAT and more cellular drug uptake [85]. In addition, increasing AA concentration in AA-LT-rHDL particles reduced intracellular lipid deposition, decreased intracellular cholesterol esters content, and DiI-oxLDL uptake, and inhibited the expressions of pro-inflammatory cytokines TNF-α and IL-6 [85]. Together, these results proved that AA modification prevented the reactivity of LT-rHDL with LCAT, thereby inhibiting the undesired drug leakage during rHDL remodeling induced by LCAT. To better fulfill the targeted-delivery of rHDL, it might be interesting to determine whether the efficacy of the incorporation of AA into LT-rHDL is better than LT-rHDL for the treatment of atherosclerosis in mice. It would also be intriguing to investigate whether the polyunsaturated fatty acids, docosahexaenoic acid (DHA) and eicosapentaenoic acid (EPA), have better efficacy than AA in preventing LCAT-induced degradation of rHDL.

## 12. Delivery of Oligonucleotides Using rHDL Nanoparticles

HDLs are highly heterogeneous and transport a large variety of lipids, proteins, and microRNAs [86]. Anti-sense nucleotides and siRNA(s) are widely used to modulate gene expression and are being considered for therapeutics of atherosclerosis [87,88,89]. One of the major issues is that the half-life of anti-sense nucleotides is usually low in the presence of serum nucleases [90]. In addition, the therapeutic efficiency of nucleic acids is relatively low owing to the non-specific bio-distribution and subsequent off-target effects of nucleotides. Recent studies demonstrate that HDLs are natural at carrying nucleotides and transporting nucleotides specifically to recipient cells [91]. Moreover, HDL-miRNA cargoes from atherosclerotic patients induced remarkable gene expression, with substantial loss of conserved mRNA targets in hepatocytes. Collectively, these results show that HDL is involved in a mechanism of intercellular communication by transporting and specific delivery of miRNAs to cells. Therefore, rHDLs are believed to be an efficient vehicle for the specific delivery of siRNA and other anti-sense nucleotides for therapeutic applications [76,92].

## 13. Molecular Imaging of rHDL-Based Nanoparticles in Atherosclerosis

Mounting evidence shows that early stages of the lesion development is dominant by monocyte recruitment followed by monocyte differentiation into macrophages in mice, whereas macrophage proliferation is more predominant in advanced atherosclerotic plaques [43,93,94]. Molecular imaging approaches are developed to detect macrophage inflammation and lipid accumulation [95,96]. Immune cells such as neutrophils and monocytes are major sources of peroxidases because these enzymes are stored in granules, such as myeloperoxidase (MPO). MPO plays important roles in the inflammatory response and perpetuation of chronic inflammation in atherosclerosis [75]. Inactivation of MPO reduced reactive oxygen species (ROS)-mediated vascular inflammation and atherosclerosis [97,98,99]. Several imaging agents targeting myeloperoxidase were developed to monitor the inflammatory response and macrophage accumulation [75,100,101,102]. rHDLs were recently developed as imaging agents due to their ability of specific delivery to macrophages [51,103]. Interestingly, superparamagnetic rHDL nanoparticles were developed for magnetically-guided drug delivery and lipoprotein drug delivery through magnetic targeting which have shown to be effective chemotherapeutic approaches for prostate cancer [104]. Recent studies demonstrate that this nanomedicine-based delivery strategy based on rHDL nanoparticles also allows for the delivery of compounds to atherosclerotic plaque. Statin-rHDL ameliorates plaque inflammation and opens a new field for atherosclerosis nanotherapy [82]. S-rHDL labeled with Cy5.5 (lipid monolayer) and DiR (hydrophobic core) show that Cy5.5 and DiR were accumulated and detected in the atherosclerotic lesions [82]. Similarly, HDL mimetic CER-001 was radiolabeled with 89Zr to allow for imaging macrophage accumulation and positron emission tomography–computed tomography (PET/CT) imaging [105].

LXRs, oxysterol-activated nuclear receptors, play an important role in RCT through promoting ABCA1 and/or ABCG1-mediated cholesterol efflux. In vivo PET imaging probes radiolabeled with zirconium-89 (89Zr) on discoidal HDL nanoparticles were made by the reconstituting apoAI and the phospholipid 1,2-dimyristoyl-sn-glycero-3-phosphocholine, the chelator deferoxamine B, and 89Zr [106]. It was demonstrated that the radioactivity in atherosclerotic aortas of rabbits was more than three-fold higher than the control animals after the injection with 89Zr-HDL nanoparticles. There was increased accumulation of radioactivity in lesions measured by the in vivo PET imaging [106]. Therefore, rHDLs demonstrated to be a reliable imaging probe and this allows us to study its in vivo properties to visualize the macrophage accumulation in advanced atherosclerotic lesions by using noninvasive PET imaging [106].

## 14. Concluding Remarks

HDL-targeted drug CETP inhibitors except anacetrapib did not decrease cardiovascular events in clinical trials. Convincing results demonstrate that increased HDL cholesterol levels do not always correlate with enhanced protective HDL properties [39,40,41], thus questioning its potential as a biomarker of HDL functionality. In addition, the association between low levels of HDL-C and CVD may be confounded by other factors, such as insulin resistance, inflammation, and/or metabolic derangements leading to altered plasma lipids. Importantly, current research is focused on both developing robust HDL functional assays and determining specific proteins or lipid molecules within the HDL complex to promote cholesterol efflux capacity for future translational and pre-clinical studies. 

Although several rHDL nanoparticles failed to regress the atherosclerotic plaques in humans, it should be noted that these clinical trials are relatively short-term studies; the duration of these trials was only 4–6 weeks. There is solid evidence that HDL beneficial effects have to do more with the achievement of a continuous flux and steady export of cholesterol, rather than absolute levels of HDL cholesterol [36]. Whether rHDL nanoparticles would be more effective for the treatment of coronary artery disease over a longer period of time remains to be investigated. Furthermore, the field of rHDL nanoparticles has developed considerably and is poised for a big leap with the application of drug delivery systems and technologies that enable the specific delivery of new compounds to the biological system [107]. In conclusion, recent advances on rHDL nanoparticles have opened up a new avenue by which to ameliorate the inflammatory response for the treatment of CVD. Better understanding of the functional roles of HDL will likely lead to new approaches to battle and monitor the expanding burden of CVD.

## Figures and Tables

**Table 1:** Reconstituted HDL nanoparticles studied in clinical trials.

Mimetic	Protein to Phospholipid Ratio	Dose Duration	Population Size	Clinical Outcomes	Reference
ETC-216	reconstituted apoAI Milano/POPC complex = 1:1.1	5 weekly infusions of ETC-216 at 45 mg/kg	*n* = 47	Modest regression of coronary plaque in the individual	[56]
MDCO-216	reconstituted apoAI Milano/POPC complex = 1:1.1	5 weekly, 20 mg/kg	(*n* = 59) placebo (*n* = 67) in statin-treated patients	Failed to produce an incremental plaque regression in statin therapy	[62]
CER-001	reconstituted human apoAI to SPM and DPPG (32:1) = 1:2.7	10 weekly, 3 mg/kg, in addition to statins	CER-001 (*n* = 135) or placebo (*n* = 137) in patients with ACS	Failed to promote regression of coronary atherosclerosis	[65]
CER-001	recombinant human apoAI to SPM and DPPG (32:1) = 1:2.7	6 weekly, 12 mg/kg	placebo *n* = 113, CER-001 *n* = 100	Failed to reduce coronary atherosclerosis on IVUS	[64]
CSL-111	human apoAI with soybean phosphatidylcholine (CSL-111)	4 weekly, 40 mg/kg, 80 mg/kg	*n* = 111	Significant improvement in the plaque characterization index	[69]
CSL-112	plasma-derived apoAI to mixed PCs isolated from soybean = 1:1.4	weekly infusions of CSL-112	Results to be concluded in 2022	CSL-112 are feasible, well tolerated	[71]

POPC: palmitoyl-oleoyl phosphatidyl choline; SPM: sphingomyelin; DPPG: dipalmitoylphosphatidyl glycerol; IVUS: intravascular ultrasonography; ACS: acute coronary syndrome; PCs: phosphatidylcholines.

## Abbreviations

AA	Arachidonic acid
ACS	Acute coronary syndrome
COX-2	Cycloxygenase-2
HDL	High-density lipoprotein
HDL-C	High-density lipoprotein cholesterol
rHDL	Reconstituted HDL
ApoAI	Apolipoprotein AI
ApoA-IM	ApoAI Milano
ApoB	Apolipoprotein B
EPA	Eicosapentaenoic acid
DHA	Docosahexaenoic acid
DPPG	Dipalmitoylphosphatidyl glycerol
HCP	Host cell proteins
HFD	High-fat diet
RCT	Reverse cholesterol transport
LCAT	Lecithin cholesterol acyltransferase
REVEAL	Randomized Evaluation of the Effects of Anacetrapib through Lipid modification
CETP	Cholesteryl ester transfer protein
CE	Cholesteryl ester
LDL	Low-density lipoprotein
ABCA1	ATP-binding cassette transporter A1
ABCG1	ATP-binding cassette transporter G1
POPC	Palmitoyl-oleoyl phosphatidyl choline
SR-BI	Scavenger receptor class B type I
SPM	Sphingomyelin
SAA	Serum amyloid A
CVD	Cardiovascular Disease
ASCVD	Atherosclerotic Cardiovascular Disease
FH	Familial hypercholesterolemia
VCAM-1	Vascular cell adhesion molecule 1
MACE	Major adverse cardiovascular events
MPO	Myeloperoxidase
MCP-1	Monocyte chemotactic factor 1
PON1	Paraoxonase1
LXR	Liver X receptors
LT	Lovastatin
BET	Bromodomain and extraterminal domain
ROS	Reactive oxygen species
I/R	Ischemic reperfusion
IVUS	Intravascular ultrasonography
QCA	Quantitative coronary angiography
PET/CT	Positron emission tomography–computed tomography
89Zr	Zirconium-89

## References

[1] Linton M.R.F., Yancey P.G., Davies S.S., Jerome W.G., Linton E.F., Song W.L., Doran A.C., Vickers K.C., Feingold K.R., Anawalt B., Boyce A., Chrousos G., Dungan K., Grossman A., Hershman J.M., Kaltsas G., Koch C., Kopp P. (2000). The Role of Lipids and Lipoproteins in Atherosclerosis. Endotext.

[2] McNamara J.J., Molot M.A., Stremple J.F., Cutting R.T. (1971). Coronary artery disease in combat casualties in Vietnam. JAMA.

[3] Stary H.C. (1989). Evolution and progression of atherosclerotic lesions in coronary arteries of children and young adults. Arteriosclerosis.

[4] Berenson G.S., Srinivasan S.R., Bao W., Newman W.P., Tracy R.E., Wattigney W.A. (1998). Association between multiple cardiovascular risk factors and atherosclerosis in children and young adults. New Engl. J. Med..

[5] Tuzcu E.M., Kapadia S.R., Tutar E., Ziada K.M., Hobbs R.E., McCarthy P.M., Young J.B., Nissen S.E. (2001). High prevalence of coronary atherosclerosis in asymptomatic teenagers and young adults: Evidence from intravascular ultrasound. Circulation.

[6] Nicholls S.J., Puri R., Anderson T., Ballantyne C.M., Cho L., Kastelein J.J., Koenig W., Somaratne R., Kassahun H., Yang J. (2016). Effect of Evolocumab on Progression of Coronary Disease in Statin-Treated Patients: The GLAGOV Randomized Clinical Trial. JAMA.

[7] Puri R., Nissen S.E., Somaratne R., Cho L., Kastelein J.J., Ballantyne C.M., Koenig W., Anderson T.J., Yang J., Kassahun H. (2016). Impact of PCSK9 inhibition on coronary atheroma progression: Rationale and design of Global Assessment of Plaque Regression with a PCSK9 Antibody as Measured by Intravascular Ultrasound (GLAGOV). Am Heart J..

[8] Galvani S., Hla T. (2017). Quality Versus Quantity: Making HDL Great Again. Arter. Thromb. Vasc. Biol..

[9] Brownell N., Rohatgi A. (2016). Modulating cholesterol efflux capacity to improve cardiovascular disease. Curr. Opin. Lipidol..

[10] Davidson W.S. (2014). HDL-C vs HDL-P: How changing one letter could make a difference in understanding the role of high-density lipoprotein in disease. Clin. Chem..

[11] Rader D.J. (2016). New Therapeutic Approaches to the Treatment of Dyslipidemia. Cell Metab..

[12] Feig J.E., Hewing B., Smith J.D., Hazen S.L., Fisher E.A. (2014). High-density lipoprotein and atherosclerosis regression: Evidence from preclinical and clinical studies. Circ. Res..

[13] Goldenberg I., Goldbourt U., Boyko V., Behar S., Reicher-Reiss H., Group B.I.P.S. (2006). Relation between on-treatment increments in serum high-density lipoprotein cholesterol levels and cardiac mortality in patients with coronary heart disease (from the Bezafibrate Infarction Prevention trial). Am. J. Cardiol..

[14] Jahagirdar R., Zhang H., Azhar S., Tobin J., Attwell S., Yu R., Wu J., McLure K.G., Hansen H.C., Wagner G.S. (2014). A novel BET bromodomain inhibitor, RVX-208, shows reduction of atherosclerosis in hyperlipidemic ApoE deficient mice. Atherosclerosis.

[15] Valenta D.T., Bulgrien J.J., Banka C.L., Curtiss L.K. (2006). Overexpression of human ApoAI transgene provides long-term atheroprotection in LDL receptor-deficient mice. Atherosclerosis.

[16] McLure K.G., Gesner E.M., Tsujikawa L., Kharenko O.A., Attwell S., Campeau E., Wasiak S., Stein A., White A., Fontano E. (2013). RVX-208, an inducer of ApoA-I in humans, is a BET bromodomain antagonist. PLoS ONE.

[17] Nicholls S.J., Gordon A., Johansson J., Wolski K., Ballantyne C.M., Kastelein J.J., Taylor A., Borgman M., Nissen S.E. (2011). Efficacy and safety of a novel oral inducer of apolipoprotein a-I synthesis in statin-treated patients with stable coronary artery disease a randomized controlled trial. J. Am. Coll. Cardiol..

[18] Nicholls S.J., Puri R., Wolski K., Ballantyne C.M., Barter P.J., Brewer H.B., Kastelein J.J., Hu B., Uno K., Kataoka Y. (2016). Effect of the BET Protein Inhibitor, RVX-208, on Progression of Coronary Atherosclerosis: Results of the Phase 2b, Randomized, Double-Blind, Multicenter, ASSURE Trial. Am. J. Cardiovasc. Drugs.

[19] Tsujikawa L.M., Fu L., Das S., Halliday C., Rakai B.D., Stotz S.C., Sarsons C.D., Gilham D., Daze E., Wasiak S. (2019). Apabetalone (RVX-208) reduces vascular inflammation in vitro and in CVD patients by a BET-dependent epigenetic mechanism. Clin. Epigenetics.

[20] Kingwell B.A., Chapman M.J., Kontush A., Miller N.E. (2014). HDL-targeted therapies: Progress, failures and future. Nat. Rev. Drug Discov..

[21] Hung A.M., Tsuchida Y., Nowak K.L., Sarkar S., Chonchol M., Whitfield V., Salas N., Dikalova A., Yancey P.G., Huang J. (2019). IL-1 Inhibition and Function of the HDL-Containing Fraction of Plasma in Patients with Stages 3 to 5 CKD. Clin. J. Am. Soc. Nephrol..

[22] Navab M., Imes S.S., Hama S.Y., Hough G.P., Ross L.A., Bork R.W., Valente A.J., Berliner J.A., Drinkwater D.C., Laks H. (1991). Monocyte transmigration induced by modification of low density lipoprotein in cocultures of human aortic wall cells is due to induction of monocyte chemotactic protein 1 synthesis and is abolished by high density lipoprotein. J. Clin. Invest..

[23] Marathe G.K., Zimmerman G.A., McIntyre T.M. (2003). Platelet-activating factor acetylhydrolase, and not paraoxonase-1, is the oxidized phospholipid hydrolase of high density lipoprotein particles. J. Biol. Chem..

[24] Kontush A., Chapman M.J. (2010). Antiatherogenic function of HDL particle subpopulations: Focus on antioxidative activities. Curr. Opin. Lipidol..

[25] Rohatgi A., de Lemos J.A., Shaul P.W. (2015). HDL cholesterol efflux capacity and cardiovascular events. New Engl. J. Med..

[26] Rohatgi A., Khera A., Berry J.D., Givens E.G., Ayers C.R., Wedin K.E., Neeland I.J., Yuhanna I.S., Rader D.R., de Lemos J.A. (2014). HDL cholesterol efflux capacity and incident cardiovascular events. New Engl. J. Med..

[27] Ebtehaj S., Gruppen E.G., Bakker S.J.L., Dullaart R.P.F., Tietge U.J.F. (2019). HDL (High-Density Lipoprotein) Cholesterol Efflux Capacity Is Associated with Incident Cardiovascular Disease in the General Population. Arter. Thromb. Vasc. Biol..

[28] Bellanger N., Orsoni A., Julia Z., Fournier N., Frisdal E., Duchene E., Bruckert E., Carrie A., Bonnefont-Rousselot D., Pirault J. (2011). Atheroprotective reverse cholesterol transport pathway is defective in familial hypercholesterolemia. Arter. Thromb. Vasc. Biol..

[29] Ogura M., Hori M., Harada-Shiba M. (2016). Association Between Cholesterol Efflux Capacity and Atherosclerotic Cardiovascular Disease in Patients with Familial Hypercholesterolemia. Arter. Throm. Vas..

[30] Huang L.H., Zinselmeyer B.H., Chang C.H., Saunders B.T., Elvington A., Baba O., Broekelmann T.J., Qi L., Rueve J.S., Swartz M.A. (2019). Interleukin-17 Drives Interstitial Entrapment of Tissue Lipoproteins in Experimental Psoriasis. Cell Metab..

[31] Huang Y., DiDonato J.A., Levison B.S., Schmitt D., Li L., Wu Y., Buffa J., Kim T., Gerstenecker G.S., Gu X. (2014). An abundant dysfunctional apolipoprotein A1 in human atheroma. Nat. Med..

[32] May-Zhang L.S., Yermalitsky V., Huang J., Pleasent T., Borja M.S., Oda M.N., Jerome W.G., Yancey P.G., Linton M.F., Davies S.S. (2018). Modification by isolevuglandins, highly reactive gamma-ketoaldehydes, deleteriously alters high-density lipoprotein structure and function. J. Biol. Chem..

[33] Mani P., Rohatgi A. (2015). Niacin Therapy, HDL Cholesterol, and Cardiovascular Disease: Is the HDL Hypothesis Defunct?. Curr. Atheroscler. Rep..

[34] Tall A.R., Rader D.J. (2018). Trials and Tribulations of CETP Inhibitors. Circ. Res..

[35] Group H.T.R.C., Bowman L., Hopewell J.C., Chen F., Wallendszus K., Stevens W., Collins R., Wiviott S.D., Cannon C.P., Braunwald E. (2017). Effects of Anacetrapib in Patients with Atherosclerotic Vascular Disease. New Engl. J. Med..

[36] Marsche G. (2015). It’s Time to Reassess the High-Density Lipoprotein (HDL) Hypothesis: CSL112, a Novel Promising Reconstituted HDL Formulation. J. Am. Heart Assoc..

[37] Rohatgi A. (2015). High-Density Lipoprotein Function Measurement in Human Studies: Focus on Cholesterol Efflux Capacity. Prog. Cardiovasc. Dis..

[38] Sacks F.M., Jensen M.K. (2018). From High-Density Lipoprotein Cholesterol to Measurements of Function: Prospects for the Development of Tests for High-Density Lipoprotein Functionality in Cardiovascular Disease. Arter. Thromb. Vasc. Biol..

[39] Zanoni P., Khetarpal S.A., Larach D.B., Hancock-Cerutti W.F., Millar J.S., Cuchel M., DerOhannessian S., Kontush A., Surendran P., Saleheen D. (2016). Rare variant in scavenger receptor BI raises HDL cholesterol and increases risk of coronary heart disease. Science.

[40] Timon-Zapata J., Laserna-Mendieta E.J., Pineda-Tenor D., Agudo-Macazaga M., Narros-Cecilia C., Rocha-Bogas M.J., Ruiz-Martin G., Gomez-Serranillos M. (2011). Extreme concentrations of high density lipoprotein cholesterol affect the calculation of low density lipoprotein cholesterol in the Friedewald formula and other proposed formulas. Clin. Biochem..

[41] Madsen C.M., Varbo A., Tybjaerg-Hansen A., Frikke-Schmidt R., Nordestgaard B.G. (2018). U-shaped relationship of HDL and risk of infectious disease: Two prospective population-based cohort studies. Eur. Heart J..

[42] Zhu L., Luu T., Emfinger C.H., Parks B.A., Shi J., Trefts E., Zeng F., Kuklenyik Z., Harris R.C., Wasserman D.H. (2018). CETP Inhibition Improves HDL Function but Leads to Fatty Liver and Insulin Resistance in CETP-Expressing Transgenic Mice on a High-Fat Diet. Diabetes.

[43] Linton M.F., Babaev V.R., Huang J., Linton E.F., Tao H., Yancey P.G. (2016). Macrophage Apoptosis and Efferocytosis in the Pathogenesis of Atherosclerosis. Circ. J..

[44] Wang D., Huang J., Gui T., Yang Y., Feng T., Tzvetkov N.T., Xu T., Gai Z., Zhou Y., Zhang J. (2020). SR-BI as a target of natural products and its significance in cancer. Semin. Cancer Biol..

[45] Ditiatkovski M., Palsson J., Chin-Dusting J., Remaley A.T., Sviridov D. (2017). Apolipoprotein A-I Mimetic Peptides: Discordance Between In Vitro and In Vivo Properties—Brief Report. Arter. Thromb. Vasc. Biol..

[46] Van Lenten B.J., Wagner A.C., Jung C.L., Ruchala P., Waring A.J., Lehrer R.I., Watson A.D., Hama S., Navab M., Anantharamaiah G.M. (2008). Anti-inflammatory apoA-I-mimetic peptides bind oxidized lipids with much higher affinity than human apoA-I. J. Lipid Res..

[47] Navab M., Anantharamaiah G.M., Reddy S.T., Hama S., Hough G., Grijalva V.R., Wagner A.C., Frank J.S., Datta G., Garber D. (2004). Oral D-4F causes formation of pre-beta high-density lipoprotein and improves high-density lipoprotein-mediated cholesterol efflux and reverse cholesterol transport from macrophages in apolipoprotein E-null mice. Circulation.

[48] Qin S., Kamanna V.S., Lai J.H., Liu T., Ganji S.H., Zhang L., Bachovchin W.W., Kashyap M.L. (2012). Reverse D4F, an apolipoprotein-AI mimetic peptide, inhibits atherosclerosis in ApoE-null mice. J. Cardiovasc Pharm. Ther..

[49] Dunbar R.L., Movva R., Bloedon L.T., Duffy D., Norris R.B., Navab M., Fogelman A.M., Rader D.J. (2017). Oral Apolipoprotein A-I Mimetic D-4F Lowers HDL-Inflammatory Index in High-Risk Patients: A First-in-Human Multiple-Dose, Randomized Controlled Trial. Clin. Transl. Sci..

[50] Marinko J.T., Huang H., Penn W.D., Capra J.A., Schlebach J.P., Sanders C.R. (2019). Folding and Misfolding of Human Membrane Proteins in Health and Disease: From Single Molecules to Cellular Proteostasis. Chem. Rev..

[51] Shah S., Chib R., Raut S., Bermudez J., Sabnis N., Duggal D., Kimball J.D., Lacko A.G., Gryczynski Z., Gryczynski I. (2016). Photophysical characterization of anticancer drug valrubicin in rHDL nanoparticles and its use as an imaging agent. J. Photochem. Photobiol. B.

[52] Zhang J., Xu D.L., Liu X.B., Bi S.J., Zhao T., Sui S.J., Ji X.P., Lu Q.H. (2016). Darapladib, a Lipoprotein-Associated Phospholipase A2 Inhibitor, Reduces Rho Kinase Activity in Atherosclerosis. Yonsei Med. J..

[53] Stewart R.A., White H.D. (2011). The role of lipoprotein-associated phospholipase a(2) as a marker and potential therapeutic target in atherosclerosis. Curr. Atheroscler. Rep..

[54] Franceschini G., Sirtori C.R., Capurso A., Weisgraber K.H., Mahley R.W. (1980). A-IMilano apoprotein. Decreased high density lipoprotein cholesterol levels with significant lipoprotein modifications and without clinical atherosclerosis in an Italian family. J. Clin. Invest..

[55] Nicholls S.J., Uno K., Kataoka Y., Nissen S.E. (2011). ETC-216 for coronary artery disease. Expert Opin. Biol. Ther..

[56] Nissen S.E., Tsunoda T., Tuzcu E.M., Schoenhagen P., Cooper C.J., Yasin M., Eaton G.M., Lauer M.A., Sheldon W.S., Grines C.L. (2003). Effect of recombinant ApoA-I Milano on coronary atherosclerosis in patients with acute coronary syndromes: A randomized controlled trial. JAMA.

[57] Ibanez B., Giannarelli C., Cimmino G., Santos-Gallego C.G., Alique M., Pinero A., Vilahur G., Fuster V., Badimon L., Badimon J.J. (2012). Recombinant HDL(Milano) exerts greater anti-inflammatory and plaque stabilizing properties than HDL(wild-type). Atherosclerosis.

[58] Reijers J.A.A., Kallend D.G., Malone K.E., Jukema J.W., Wijngaard P.L.J., Burggraaf J., Moerland M. (2017). MDCO-216 Does Not Induce Adverse Immunostimulation, in Contrast to Its Predecessor ETC-216. Cardiovasc. Drugs Ther..

[59] Kempen H.J., Schranz D.B., Asztalos B.F., Otvos J., Jeyarajah E., Drazul-Schrader D., Collins H.L., Adelman S.J., Wijngaard P.L. (2014). Incubation of MDCO-216 (ApoA-IMilano/POPC) with Human Serum Potentiates ABCA1-Mediated Cholesterol Efflux Capacity, Generates New Prebeta-1 HDL, and Causes an Increase in HDL Size. J. Lipids.

[60] Kempen H.J., Asztalos B.F., Moerland M., Jeyarajah E., Otvos J., Kallend D.G., Bellibas S.E., Wijngaard P.L. (2016). High-Density Lipoprotein Subfractions and Cholesterol Efflux Capacities After Infusion of MDCO-216 (Apolipoprotein A-IMilano/Palmitoyl-Oleoyl-Phosphatidylcholine) in Healthy Volunteers and Stable Coronary Artery Disease Patients. Arter. Thromb. Vasc. Biol..

[61] Kallend D.G., Reijers J.A., Bellibas S.E., Bobillier A., Kempen H., Burggraaf J., Moerland M., Wijngaard P.L. (2016). A single infusion of MDCO-216 (ApoA-1 Milano/POPC) increases ABCA1-mediated cholesterol efflux and pre-beta 1 HDL in healthy volunteers and patients with stable coronary artery disease. Eur. Heart J. Cardiovasc. Pharmacother..

[62] Nicholls S.J., Puri R., Ballantyne C.M., Jukema J.W., Kastelein J.J.P., Koenig W., Wright R.S., Kallend D., Wijngaard P., Borgman M. (2018). Effect of Infusion of High-Density Lipoprotein Mimetic Containing Recombinant Apolipoprotein A-I Milano on Coronary Disease in Patients With an Acute Coronary Syndrome in the MILANO-PILOT Trial: A Randomized Clinical Trial. Jama Cardiol..

[63] Tardy C., Goffinet M., Boubekeur N., Ackermann R., Sy G., Bluteau A., Cholez G., Keyserling C., Lalwani N., Paolini J.F. (2014). CER-001, a HDL-mimetic, stimulates the reverse lipid transport and atherosclerosis regression in high cholesterol diet-fed LDL-receptor deficient mice. Atherosclerosis.

[64] Tardif J.C., Ballantyne C.M., Barter P., Dasseux J.L., Fayad Z.A., Guertin M.C., Kastelein J.J., Keyserling C., Klepp H., Koenig W. (2014). Effects of the high-density lipoprotein mimetic agent CER-001 on coronary atherosclerosis in patients with acute coronary syndromes: A randomized trial. Eur. Heart J..

[65] Nicholls S.J., Andrews J., Kastelein J.J.P., Merkely B., Nissen S.E., Ray K.K., Schwartz G.G., Worthley S.G., Keyserling C., Dasseux J.L. (2018). Effect of Serial Infusions of CER-001, a Pre-beta High-Density Lipoprotein Mimetic, on Coronary Atherosclerosis in Patients Following Acute Coronary Syndromes in the CER-001 Atherosclerosis Regression Acute Coronary Syndrome Trial: A Randomized Clinical Trial. JAMA Cardiol..

[66] Richart A., Reddy M., Natoli A., Heywood S., Khalaji M., Lancaster G., Diditchenko S., Navdaev A., Kingwell B. (2019). ApoA-I nanoparticles (CSL-111) Directly Modulates Inflammatory Cells After Myocardial Infarction in Mice. Arterioscler. Thromb. Vasc. Biol..

[67] Bhushan S., Kondo K., Predmore B.L., Zlatopolsky M., King A.L., Pearce C., Huang H., Tao Y.X., Condit M.E., Lefer D.J. (2012). Selective beta2-adrenoreceptor stimulation attenuates myocardial cell death and preserves cardiac function after ischemia-reperfusion injury. Arterioscler. Thromb. Vasc. Biol..

[68] Bhushan S., Kondo K., Polhemus D.J., Otsuka H., Nicholson C.K., Tao Y.X., Huang H., Georgiopoulou V.V., Murohara T., Calvert J.W. (2014). Nitrite therapy improves left ventricular function during heart failure via restoration of nitric oxide-mediated cytoprotective signaling. Circ. Res..

[69] Tardif J.C., Gregoire J., L’Allier P.L., Ibrahim R., Lesperance J., Heinonen T.M., Kouz S., Berry C., Basser R., Lavoie M.A. (2007). Effects of reconstituted high-density lipoprotein infusions on coronary atherosclerosis: A randomized controlled trial. JAMA.

[70] Diditchenko S., Gille A., Pragst I., Stadler D., Waelchli M., Hamilton R., Leis A., Wright S.D. (2013). Novel formulation of a reconstituted high-density lipoprotein (CSL112) dramatically enhances ABCA1-dependent cholesterol efflux. Arter. Thromb. Vasc. Biol..

[71] Waksman R., Torguson R., Kent K.M., Pichard A.D., Suddath W.O., Satler L.F., Martin B.D., Perlman T.J., Maltais J.A., Weissman N.J. (2010). A first-in-man, randomized, placebo-controlled study to evaluate the safety and feasibility of autologous delipidated high-density lipoprotein plasma infusions in patients with acute coronary syndrome. J. Am. Coll. Cardiol..

[72] Marsche G., Saemann M.D., Heinemann A., Holzer M. (2013). Inflammation alters HDL composition and function: Implications for HDL-raising therapies. Pharm. Ther..

[73] Han C.Y., Tang C., Guevara M.E., Wei H., Wietecha T., Shao B., Subramanian S., Omer M., Wang S., O’Brien K.D. (2016). Serum amyloid A impairs the antiinflammatory properties of HDL. J. Clin. Invest..

[74] Birner-Gruenberger R., Schittmayer M., Holzer M., Marsche G. (2014). Understanding high-density lipoprotein function in disease: Recent advances in proteomics unravel the complexity of its composition and biology. Prog. Lipid Res..

[75] Huang J., Milton A., Arnold R.D., Huang H., Smith F., Panizzi J.R., Panizzi P. (2016). Methods for measuring myeloperoxidase activity toward assessing inhibitor efficacy in living systems. J. Leukoc. Biol..

[76] Lacko A.G., Sabnis N.A., Nagarajan B., McConathy W.J. (2015). HDL as a drug and nucleic acid delivery vehicle. Front. Pharm..

[77] Raut S., Mooberry L., Sabnis N., Garud A., Dossou A.S., Lacko A. (2018). Reconstituted HDL: Drug Delivery Platform for Overcoming Biological Barriers to Cancer Therapy. Front. Pharm..

[78] Chaudhary J., Bower J., Corbin I.R. (2019). Lipoprotein Drug Delivery Vehicles for Cancer: Rationale and Reason. Int. J. Mol. Sci..

[79] Zhang M., He J., Jiang C., Zhang W., Yang Y., Wang Z., Liu J. (2017). Plaque-hyaluronidase-responsive high-density-lipoprotein-mimetic nanoparticles for multistage intimal-macrophage-targeted drug delivery and enhanced anti-atherosclerotic therapy. Int. J. Nanomed..

[80] Yu M., Amengual J., Menon A., Kamaly N., Zhou F., Xu X., Saw P.E., Lee S.J., Si K., Ortega C.A. (2017). Targeted Nanotherapeutics Encapsulating Liver X Receptor Agonist GW3965 Enhance Antiatherogenic Effects without Adverse Effects on Hepatic Lipid Metabolism in Ldlr(−/−) Mice. Adv. Healthc. Mater..

[81] Tang J., Baxter S., Menon A., Alaarg A., Sanchez-Gaytan B.L., Fay F., Zhao Y., Ouimet M., Braza M.S., Longo V.A. (2016). Immune cell screening of a nanoparticle library improves atherosclerosis therapy. Proc. Natl. Acad. Sci. USA.

[82] Duivenvoorden R., Tang J., Cormode D.P., Mieszawska A.J., Izquierdo-Garcia D., Ozcan C., Otten M.J., Zaidi N., Lobatto M.E., van Rijs S.M. (2014). A statin-loaded reconstituted high-density lipoprotein nanoparticle inhibits atherosclerotic plaque inflammation. Nat. Commun..

[83] Tang J., Lobatto M.E., Hassing L., van der Staay S., van Rijs S.M., Calcagno C., Braza M.S., Baxter S., Fay F., Sanchez-Gaytan B.L. (2015). Inhibiting macrophage proliferation suppresses atherosclerotic plaque inflammation. Sci. Adv..

[84] Guo Y., Yuan W., Yu B., Kuai R., Hu W., Morin E.E., Garcia-Barrio M.T., Zhang J., Moon J.J., Schwendeman A. (2018). Synthetic High-Density Lipoprotein-Mediated Targeted Delivery of Liver X Receptors Agonist Promotes Atherosclerosis Regression. EBioMedicine.

[85] He H., Liu L., Bai H., Wang J., Zhang Y., Zhang W., Zhang M., Wu Z., Liu J. (2014). Arachidonic acid-modified lovastatin discoidal reconstituted high density lipoprotein markedly decreases the drug leakage during the remodeling behaviors induced by lecithin cholesterol acyltransferase. Pharm. Res..

[86] Michell D.L., Vickers K.C. (2016). HDL and microRNA therapeutics in cardiovascular disease. Pharm. Ther..

[87] Graham M.J., Lee R.G., Brandt T.A., Tai L.J., Fu W., Peralta R., Yu R., Hurh E., Paz E., McEvoy B.W. (2017). Cardiovascular and Metabolic Effects of ANGPTL3 Antisense Oligonucleotides. New Engl. J. Med..

[88] Lim G.B. (2017). Dyslipidaemia: ANGPTL3: A therapeutic target for atherosclerosis. Nat. Rev. Cardiol..

[89] Wang D., Atanasov A.G. (2019). The microRNAs Regulating Vascular Smooth Muscle Cell Proliferation: A Minireview. Int. J. Mol. Sci..

[90] Davalos A., Chroni A. (2015). Antisense oligonucleotides, microRNAs, and antibodies. Handb. Exp. Pharm..

[91] Tabet F., Vickers K.C., Cuesta Torres L.F., Wiese C.B., Shoucri B.M., Lambert G., Catherinet C., Prado-Lourenco L., Levin M.G., Thacker S. (2014). HDL-transferred microRNA-223 regulates ICAM-1 expression in endothelial cells. Nat. Commun..

[92] Raut S., Dasseux J.L., Sabnis N.A., Mooberry L., Lacko A. (2018). Lipoproteins for therapeutic delivery: Recent advances and future opportunities. Ther. Deliv..

[93] Kanter J.E. (2017). Monocyte Recruitment Versus Macrophage Proliferation in Atherosclerosis. Circ. Res..

[94] Babaev V.R., Huang J., Ding L., Zhang Y., May J.M., Linton M.F. (2018). Loss of Rictor in Monocyte/Macrophages Suppresses Their Proliferation and Viability Reducing Atherosclerosis in LDLR Null Mice. Front. Immunol..

[95] DelBove C.E., Strothman C.E., Lazarenko R.M., Huang H., Sanders C.R., Zhang Q. (2019). Reciprocal modulation between amyloid precursor protein and synaptic membrane cholesterol revealed by live cell imaging. Neurobiol. Dis..

[96] Wildgruber M., Swirski F.K., Zernecke A. (2013). Molecular imaging of inflammation in atherosclerosis. Theranostics.

[97] Huang J., Smith F., Panizzi J.R., Goodwin D.C., Panizzi P. (2015). Inactivation of myeloperoxidase by benzoic acid hydrazide. Arch. Biochem. Biophys..

[98] Huang J., Smith F., Panizzi P. (2014). Ordered cleavage of myeloperoxidase ester bonds releases active site heme leading to inactivation of myeloperoxidase by benzoic acid hydrazide analogs. Arch. Biochem. Biophys..

[99] Cheng D., Talib J., Stanley C.P., Rashid I., Michaelsson E., Lindstedt E.L., Croft K.D., Kettle A.J., Maghzal G.J., Stocker R. (2019). Inhibition of MPO (Myeloperoxidase) Attenuates Endothelial Dysfunction in Mouse Models of Vascular Inflammation and Atherosclerosis. Arter. Thromb. Vasc. Biol..

[100] Ronald J.A., Chen J.W., Chen Y., Hamilton A.M., Rodriguez E., Reynolds F., Hegele R.A., Rogers K.A., Querol M., Bogdanov A. (2009). Enzyme-sensitive magnetic resonance imaging targeting myeloperoxidase identifies active inflammation in experimental rabbit atherosclerotic plaques. Circulation.

[101] Breckwoldt M.O., Chen J.W., Stangenberg L., Aikawa E., Rodriguez E., Qiu S., Moskowitz M.A., Weissleder R. (2008). Tracking the inflammatory response in stroke in vivo by sensing the enzyme myeloperoxidase. Proc. Natl. Acad. Sci. USA.

[102] Chen J.W., Breckwoldt M.O., Aikawa E., Chiang G., Weissleder R. (2008). Myeloperoxidase-targeted imaging of active inflammatory lesions in murine experimental autoimmune encephalomyelitis. Brain.

[103] Sanchez-Gaytan B.L., Fay F., Lobatto M.E., Tang J., Ouimet M., Kim Y., van der Staay S.E., van Rijs S.M., Priem B., Zhang L. (2015). HDL-mimetic PLGA nanoparticle to target atherosclerosis plaque macrophages. Bioconjug. Chem..

[104] Sabnis S., Sabnis N.A., Raut S., Lacko A.G. (2017). Superparamagnetic reconstituted high-density lipoprotein nanocarriers for magnetically guided drug delivery. Int. J. Nanomed..

[105] Zheng K.H., van der Valk F.M., Smits L.P., Sandberg M., Dasseux J.L., Baron R., Barbaras R., Keyserling C., Coolen B.F., Nederveen A.J. (2016). HDL mimetic CER-001 targets atherosclerotic plaques in patients. Atherosclerosis.

[106] Perez-Medina C., Tang J., Abdel-Atti D., Hogstad B., Merad M., Fisher E.A., Fayad Z.A., Lewis J.S., Mulder W.J., Reiner T. (2015). PET Imaging of Tumor-Associated Macrophages with 89Zr-Labeled High-Density Lipoprotein Nanoparticles. J. Nucl. Med..

[107] Flores A.M., Ye J., Jarr K.U., Hosseini-Nassab N., Smith B.R., Leeper N.J. (2019). Nanoparticle Therapy for Vascular Diseases. Arter. Thromb. Vasc. Biol..

